# Supplementary material for: The Effect of Donors’ Demographic Characteristics in Renal Function Post-Living Kidney Donation. Analysis of a UK Single Centre Cohort
Source: J Clin Med. 2019 Jun 20;8(6):883. doi: 10.3390/jcm8060883 (PMC6616400; doi:10.3390/jcm8060883)
Supplement: Supplementary file 1 [file jcm-08-00883-s001.pdf]

Timeline	Age			WHO classification				Sex			Ethnicity				Genetic Relationship		
	< 60 years	> or = 60 years	*p*	BMI < 25	25 < BMI ≤ 29	BMI ≥ 30	*p*	Female	Male	*p*	Asian	African	Caucasian	*p*	LRD	LURD	*p*
Donation	**93 ± 24**	80 ± 39	**<0.001**	92 ± 29	91 ± 30	89 ± 20	0.495	87 ± 22	**95 ± 32**	**<.001**	92 ± 24	**103 ± 23**	87 ± 28	**<0.001**	91 ± 26	90 ± 29	0.948
6 months	**67 ± 16**	52 ± 10	**<0.001**	65 ± 16	63 ± 16	64 ± 16	0.482	64 ± 16	65 ± 16	0.441	**66 ± 17**	**76 ± 17**	61 ± 14	**<0.001**	64 ± 17	64 ± 15	0.787
12 months	**67 ± 16**	52 ± 11	**<0.001**	65 ± 15	63 ± 16	66 ± 17	0.219	64 ± 16	65 ± 17	0.759	**67 ± 18**	**75 ± 18**	61 ± 14	**<0.001**	64 ± 17	65 ± 15	0.735
24 months	**69 ± 16**	53 ± 11	**<0.001**	66 ± 16	65 ± 17	66 ± 16	0.750	66 ± 17	67 ± 16	0.803	**69 ± 18**	**76 ± 18**	63 ± 15	**<0.001**	65 ± 17	67 ± 16	0.245
36 months	**70 ± 17**	55 ± 10	**<0.001**	69 ± 16	66 ± 17	67 ± 15	0.419	67 ± 17	67 ± 16	0.809	**71 ± 18**	**78 ± 20**	64 ± 14	**<.001**	66 ± 17	69 ± 16	0.054
48 months	**72 ± 18**	54 ± 11	**<0.001**	71 ± 18	68 ± 18	67 ± 16	0.220	69 ± 19	68 ± 17	0.542	**73 ± 20**	**77 ± 19**	65 ± 16	**<.001**	68 ± 18	70 ± 17	0.184
60 months	**72 ± 18**	56 ± 11	**<0.001**	71 ± 17	69 ± 18	69 ± 17	0.674	70 ± 17	70 ± 18	0.760	**73 ± 19**	**77 ± 20**	68 ± 16	**0.002**	69 ± 18	71 ± 17	0.167
Δ eGFR 6 months	.26 ± .16	**.31 ± .15**	**0.003**	.27 ± .15	.28 ± .15	.26 ± .17	0.420	.25 ± .16	**.30 ± .15**	**<0.001**	.27 ± .15	.23 ± .18	**.28 ± .15**	**0.035**	.28 ± .15	.26 ± .16	0.129
Δ eGFR 12 months	.25 ± .15	**.30 ± .17**	**0.005**	.25 ± .14	**.28 ± .15**	.24 ± .16	**0.015**	.24 ± .15	**.29 ± .15**	**<0.001**	.26 ± .14	.24 ± .17	.27 ± .15	0.339	.27 ± .15	.25 ± .16	0.153
Δ eGFR 24 months	.23 ± .14	**.27 ± .18**	**0.026**	.24 ± .15	.25 ± .15	.22 ± .15	0.173	.22 ± .15	**.26 ± .16**	**0.002**	.24 ± .15	.20 ± 17	.24 ± .15	0.098	.24 ± .15	.23 ± .15	0.222
Δ eGFR 36 months	.21 ± .16	.25 ± .19	0.59	.21 ± .16	.23 ± .17	.21 ± .15	0.223	.20 ± .16	**.24 ± .16**	**0.003**	.21 ± .16	.19 ± .15	.23 ± .16	0.295	**.23 ± .16**	.20 ± .17	**0.030**
Δ eGFR 48 months	.19 ± .17	**.26 ± .18**	**0.001**	.17 ± .18	.22 ± .17	.20 ± .16	0.107	.17 ± .18	**.24 ± .16**	**<0.001**	.18 ± .16	.20 ± .16	.20 ± .18	0.662	.20 ± .18	.19 ± .16	0.454
Δ eGFR 60 months	.17 ± .17	**.23 ± .17**	**0.014**	.16 ± .17	.19 ± .17	.17 ± .17	0.330	.14 ± .17	**.22 ± .16**	**<0.001**	.17 ± .15	.16 ± .17	.18 ± .17	0.752	.19 ± .17	.16 ± .17	0.129

Appendix A. Mean eGFR during follow up and percentual difference between mean eGFR during follow up and eGFR at donation (Δ eGFR). Mean ± SD eGFR reported in ml/min/1.73m^2. In bold are highlighted statistically significant higher values.
